# Supplementary material for: Reconciling Mining with the Conservation of Cave Biodiversity: A Quantitative Baseline to Help Establish Conservation Priorities
Source: PLoS One. 2016 Dec 20;11(12):e0168348. doi: 10.1371/journal.pone.0168348 (PMC5173368; doi:10.1371/journal.pone.0168348)
Supplement: S1 Dataset — (ZIP) [file pone.0168348.s002.zip › Taxa/Serra Norte/SN_2013/N4WS/Lista N4WS-30.pdf]

CAVIDADE N4WS-0030

Classe	Ordem	Fam/Outros	Gên/Outros	Espécie	S-U	U-S	Zon.
Arachnida	Acari	(Oribatida)		sp.4	X		E
Arachnida	Amblypygi	Charinidae	*Charinus*	jovem		1	E
Arachnida	Araneae	Araneidae	*Micrathena*	sp.1	X		E
Arachnida	Araneae	Barychelidae		jovem	1		E
Arachnida	Araneae	Ctenidae		jovem	1		E
Arachnida	Araneae	Filistatidae		jovem		X	E
Arachnida	Araneae	Ochyroceratidae		jovem		X	E
Arachnida	Araneae	Pholcidae	*Leptopholcus*	sp.1	X	X	E
Arachnida	Araneae	Pholcidae	*Mesabolivar*	*eberhard*	X	X	E
Arachnida	Araneae	Pholcidae	Ninetinae	sp.1	X	X	E
Arachnida	Araneae	Salticidae	*Amphidraus*	sp.1		X	E
Arachnida	Araneae	Tetragnathidae		jovem	X		E
Entognatha	Collembola	Entomobryidae		sp.1	X		E
Entognatha	Collembola	Entomobryidae		sp.3	X		E
Entognatha	Collembola	Entomobryidae		sp.6		X	E
Entognatha	Collembola	Paronellidae		sp.1	X	X	E
Entognatha	Collembola	Paronellidae		sp.6	X		E
Insecta	Blattodea	Blattellidae		jovem	1		E
Insecta	Coleoptera	Nitidulidae		sp.2		X	E
Insecta	Diptera	Cecidomyiidae	Cecidomyiinae	sp.	X		E
Insecta	Diptera	Ceratopogonidae		sp.	X		E
Insecta	Diptera	Culicidae	*Culex*	sp.		X	E
Insecta	Diptera	Drosophilidae	*Drosophila*	*eleonorae*		X	E
Insecta	Diptera	Drosophilidae		sp.	X		E
Insecta	Diptera	Psychodidae	*Sciopemyia*	*sordellii*		X	E
Insecta	Diptera	Sarcophagidae		sp.		X	E
Insecta	Diptera	Tipulidae	Tipulinae	sp.	X		E
Insecta	Heteroptera	Miridae		jovem		X	E
Insecta	Heteroptera	Reduviidae	Emesinae	sp.1		1	E
Insecta	Homoptera	Derbidae		sp.1	X		E
Insecta	Hymenoptera	Chalcidoidea		sp.1		X	E
Insecta	Hymenoptera	Formicidae	*Apterostigma*	sp.1	X		E
Insecta	Hymenoptera	Formicidae	*Camponotus*	sp.1	X		E
Insecta	Hymenoptera	Formicidae	*Hypoponera*	sp.1		X	E
Insecta	Hymenoptera	Formicidae	*Nylanderia*	sp.2	X		E
Insecta	Hymenoptera	Formicidae	*Pheidole*	sp.2	X		E
Insecta	Hymenoptera	Formicidae	*Wasmannia*	sp.1	X		E
Insecta	Isoptera			sp.		X	E
Insecta	Orthoptera	Phalangopsidae	*Paraclodes*	sp.	8		E
Insecta	Psocoptera	Psocomorpha		jovem		X	E
Insecta	Psocoptera	Psyllipsocidae	*Psyllipsocus*	sp.2		X	E
Insecta	Thysanoptera	Thripidae		sp.1		X	E
Amphibia	Anura	Strabomantidae	*Pristimantis*	cf. *fenestratus*	4	2	E
Mammalia	Chiroptera	Emballonuridae	*Peropteryx*	sp.	4	7	E
